# Prostate positioning errors associated with two automatic registration based image guidance strategies

**DOI:** 10.1120/jacmp.v10i4.3071

**Published:** 2009-10-15

**Authors:** D. Ryan, C. Rivest, Terence A. Riauka, Albert D. Murtha, B. Gino Fallone

**Affiliations:** ^1^ Department of Physics University of Alberta Edmonton Alberta Canada; ^2^ Department of Oncology University of Alberta Edmonton Alberta Canada; ^3^ Department of Medical Physics Cross Cancer Institute Edmonton Alberta Canada; ^4^ Department of Radiation Oncology Cross Cancer Institute Edmonton Alberta Canada

**Keywords:** helical tomotherapy, prostate motion, image registration

## Abstract

Daily image guidance for helical tomotherapy prostate patients is based on the registration of pretreatment megavoltage CT (MVCT) images and the original planning CT. The goal of registration, whether manual or automatic, is the overlap of the prostate; otherwise prostate misplacement may compromise the efficacy of treatment or lead to increased toxicity. A previous study demonstrated that without the aid of implanted fiducials, manual registration results in inaccurate prostate positioning. The objective of this work is to quantify prostate misplacement that results from automatic bone matching (BM) and image matching (IM) registration algorithms. 204 MVCT images from eight high‐risk tomotherapy prostate patients were incorporated into this retrospective study. BM and IM registration algorithms – based on maximization of mutual information of bony anatomy only and the entire image, respectively – were used to independently register MVCT images to their respective planning images. A correlation coefficient based algorithm that uses known planning CT contour information was used for automatic prostate localization in each MVCT image. Daily prostate misplacement was determined by repositioning as calculated from the BM and the IM algorithms. Mean (±SD) and maximum 3D prostate positioning errors were 3.7±2.1mm and 11.8 mm for bone matching, and 4.6±2.3mm and 11.5 mm for image matching. In terms of translational directions, IM would lead to prostate positioning error ≥3mm in any of the LR, AP or SI directions in 62% of treatment fractions. The corresponding value for BM is 51%. The values for positioning errors ≥5mm were 29% and 17% for IM and BM, respectively. This data suggests automatic daily image guidance for tomotherapy prostate patients should be based on bone matching instead of image matching.

PACS number: 87.19.xj, 87.57.nj

## I. INTRODUCTION

Movement of the prostate gland throughout a course of radiation therapy occurs as a result of daily variations in bladder and rectal filling.^(^ ^1^ ^,^ ^2^ ^,^ ^3^ ^,^ ^4^ ^)^ This movement can result in significant changes in the radiation dose received by the prostate and adjacent critical structures.^(^ ^5^ ^,^ ^6^ ^)^ As treatments evolve and use more aggressive or complex treatment approaches (i.e. dose escalation, hypofractionation margin reduction, or dominant nodule boost), the need to correct for daily setup errors of the prostate will take on greater importance. Daily image guidance on the Hi·Art II system (TomoTherapy, Inc. Madison, WI) provides a solution to correct for prostate motion.^(7)^ After positioning the patient on the treatment couch, a three‐dimensional (3D) mega‐voltage CT (MVCT) image of the patient in the treatment position is acquired. The image is subsequently registered to the patient's planning CT and, based on the rigid registration, the patient is repositioned and treated. In our clinic, the goal of registration, whether manual or automated, is an overlap of the prostate gland in the MVCT and planning CT images to ensure that after patient repositioning the target volume is in the same geometric position on a day‐today basis. Otherwise, patient repositioning will result in prostate misplacement during treatment delivery and the efficacy of the treatment may be compromised.

The Hi·Art II system has both manual and automatic capabilities for registering daily MVCT and planning CT images. Automatic registration allows the user to select whether the registration is based on either the bony anatomy, the bone plus tissue, or the entire image. The system does not have the ability to perform automatic registration based on the overlap of a particular region of interest (ROI), such as the prostate. Alignment of the prostate gland in MVCT images and planning CT images using manual registration techniques has been demonstrated to result in inaccurate daily positioning of the prostate.^(8)^

The objective of this work is to determine whether daily patient repositioning of tomotherapy prostate patients based on automatic registration of bony anatomy or automatic registration of the entire image will lead to the correct positioning of the prostate on a day‐to‐day basis. In order to examine this question, accurate prostate localization in daily MVCT images is required. Traditionally, prostate localization in treatment images has been performed through either contouring of the prostate^(9)^ or with the use of implanted fiducials.^(10)^ The inherent reduced contrast of MVCT images compared to kVCT images makes contouring difficult and subject to observer variability, while implantation of fiducials is invasive and not practical for all patients. Recently, a registration‐based technique that uses contour information from an initial kVCT to automatically localize the prostate in subsequent CT images has been developed and validated.^(^ ^11^ ^,^ ^12^ ^,^ ^13^ ^)^ We have produced our own software based on this technique that uses median filtration in order to accurately and automatically localize the prostate in patient MVCT images. The software was used to retrospectively localize the prostate in patient treatment images acquired on the Hi·Art II system. In addition, we used established automatic voxel‐based methods to register either the bony anatomy or all voxels in MVCT and planning CT images.^(^ ^14^ ^,^ ^15^ ^)^ By evaluating automatic registration and prostate localization results, we calculated the prostate misplacement that would have occurred if daily patient repositioning was based on automatic registration of bony anatomy or if it was based on automatic registration of the entire image.

## II. MATERIALS AND METHODS

### A. Patient images

Images from eight random research patients undergoing prostate treatment on the Hi·Art II system were used for this retrospective study. The planning CT images were acquired on a Philips PQ5000 scanner (Philips Medical Systems, Cleveland, OH), while daily MVCT images were acquired prior to each treatment fraction on the TomoTherapy Hi·Art II unit. The sole requirement in the MVCT acquisition protocol mandated that the midplane of the prostate be captured; as such, the extent of the patient imaged in the superior/inferior direction varied from fraction to fraction, but was typically on the order of approximately 15 cm. Of the eight patients, six patients underwent 25 treatment fractions, one received 23, and the final patient had 31 fractions. Each of the 204 MVCT images was manually registered to their respective planning CT image on the Reveal‐MVS Fusion System (Mirada Solutions Limited, Oxford Centre for Innovation, Oxford, UK) to account for any gross misalignments that may have occurred as a result of poor initial patient positioning. The manual registrations were performed by a single physicist with the objective of achieving sub‐cm accuracy, well within the capture ranges of the automatic software used in the study.

### B. Automatic registration

Using the manual results as the initial alignment, each MVCT image was registered to its respective planning CT using two different automatic rigid algorithms integrated in our in‐house registration software. One algorithm is designed to produce the best overall alignment of the registered images, while the other achieves alignment of just the bony anatomy. These registration methods will be referred to as image matching (IM) and bone matching (BM) throughout this paper, respectively. Both voxel‐based algorithms use the Nelder‐Mead simplex optimizer^(16)^ to maximize different adaptations of the mutual information cost function proposed by Mattes et al.^(15)^ For IM, all planning CT image voxels were used in calculating the mutual information metric; for BM, only the planning CT voxels corresponding to bone were included, an idea first proposed by Ruchala et al.^(14)^ As such, the voxels used for BM, which were segmented via thresholding, represent a subset of the voxels used for IM. The registration software was developed using the Insight Segmentation and Registration Toolkit (ITK),^(17)^ an open‐source, object‐oriented software package. The toolkit consists of a collection of C++ classes designed for image processing, segmentation, and registration that can be implemented in user‐developed software.

### C. Automatic prostate localization

Court and Dong^(11)^ demonstrated that automatic localization of the prostate on treatment CT images could be achieved by registering treatment images to a planning CT image using only the voxels in the planning CT gross tumor volume (GTV) plus a 3 mm border, or expansion margin, when calculating the registration cost function. In their implementation, the mean absolute difference (MAD) between overlapping voxels was minimized. They assumed translations only, neglecting any rotation and changes in the size and shape of the prostate. Smitsmans et al.^(12)^ incorporated prostate rotation into the algorithm's design and investigated its capabilities when other cost functions instead of MAD were used. More recently, they validated the technique for use on kV cone beam CT (CBCT) images as well.^(13)^ We propose using this registration‐based method for localizing the prostate in MVCT images acquired on the TomoTherapy's High·Art II system.

Our implementation uses the correlation coefficient metric and only radiation oncologist‐delineated prostate planning CT voxels plus a small border are used for calculation of the cost function. Voxels in the border region corresponding to intestinal gas or bone are filtered out by thresholding, in order to reduce their influence on registration. The increased noise and reduced contrast in MVCT images relative to conventional kVCT and kV CBCT images make application of the technique to our situation nontrivial. To reduce the complexity of the problem, we followed Court and Dong's model in that only prostate translation is assumed. In addition, a noise reducing median filter was applied to MVCT images as a preprocessing procedure prior to each registration.

Although the prostate localization technique has been thoroughly validated for other treatment CT modalities, it had not been applied to MVCT images. Therefore, we believed that verification of its efficacy was required. This task was carried out using images from two patients, separate from the eight patients described above, who had three localization seeds implanted in the prostate. An initial planning CT and a single MVCT were acquired for each patient and the prostate was contoured in the planning CT. The true motion of the prostate was determined by analyzing the center of gravity (COG) of the seeds in each image. The seeds were subsequently digitally removed by editing the image intensity data^(18)^ prior to registration with the prostate localization software. The dependence of the software on the border surrounding the contoured prostate was established by repeating the registrations with 1 mm interval borders ranging from 0 to 10 mm. The improvements achieved by incorporating median filtration into the technique were verified by repeating the registrations without use of the filter. Registration results were compared to the known prostate motion as determined by the seed positions. We also investigated the algorithm's dependence on the radiation oncologist's delineation of the prostate. One patient was randomly selected and the radiation oncologist was asked to recontour the planning CT prostate months after the original contour had been drawn. Ten MVCT images for that patient were chosen at random and automatic prostate localization was repeated using the new contour for selection of the planning CT voxels used in registration.

### D. Multistart procedure

To reduce registration uncertainty and eliminate any gross mis‐registrations, a multi‐start optimization procedure was employed.^(19)^ Every registration was repeated ten times, each time with a different random initial image overlap. For bone and image matching, the MVCT image position was randomly offset from the manual alignment position by a maximum of 10 mm and 5° in 3D Euclidean space. In the prostate localization procedure, the initial MVCT image position was randomly offset from the optimal bone matching alignment by a maximum of 5 mm. Of the ten starts, the registration that resulted in the optimal mutual information value for IM and BM, and the optimal correlation coefficient value for prostate localization, was chosen as the true result. A simple test was implemented to reduce the possibility that the optimal cost function value corresponded to a false minimum. If more than two of the other nine registrations differed from the optimal result by greater than a 1 mm translation or 1° rotation in 3D Euclidean space, the result was discarded and the entire multistart optimization procedure was repeated. If the multistart procedure failed a second time, that registration was not included in subsequent analysis. In addition, all results were visually inspected and any registration that failed visual inspection was also repeated.

## III. RESULTS

### A. Prostate localization validation

**Figure 1:**
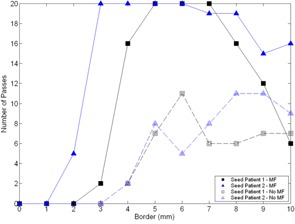
Number of passes as a function of the contour border used in the registration cost function calculation for the two test patient image pairs with implanted prostate localization seeds, with and without use of a median filter (MF). Each multistart procedure consisted of 20 starts, and a registration was considered a pass if the prostate motion was within half a planning CT voxel of the true motion in each of the three lateral directions.

A simple pass/fail criterion was used to ensure the prostate localization algorithm validated for other treatment CT imaging modalities could also be used on MVCT images. The multistart procedure described above was carried out for each image pair that contained implanted fiducials using each of the different border dimensions, with and without median filtration. To improve statistics, twenty random starts were used instead of ten. Registrations that localized the prostate within half a planning CT voxel to its true position in each translational direction as determined by the implanted seeds were considered a pass. This corresponds to accuracy within 0.94 mm, 0.94 mm, and 1.50 mm in the lateral left/right (LR), anterior/posterior (AP), and superior/inferior (SI) directions, respectively. The number of passes for each multistart procedure is plotted in Fig. 1. The improved efficacy of the algorithm when median filtration is used is evident by the data. Focusing on the median filtered results, a dependence on the prostate border can be established. Results suggest that having too large or too small a prostate border when calculating the correlation coefficient cost function can lead to erroneous results. These results can be explained if we consider the inherent information contained in the voxels used in the calculation of the correlation coefficient metric. The individual voxels in the prostate itself have a fairly uniform range of intensity values in CT images. This lack of information makes it very difficult for the registration algorithm to accurately converge when little or no border is used and when prostate voxels themselves contribute to the vast majority or all of the voxels used when calculating the registration cost function. On the other hand, as more and more surrounding voxels are included, the contribution of the prostate itself to the cost function becomes diminished and the registration result slowly diverges from the truth. The competing effects set both a lower and upper limit on the border size, and it was decided that a 6 mm border should be used throughout the remainder of this work. The true prostate motion between MVCT and planning CT images for the two test patients are compared with the values determined by registration in Table 1. Deviations between registration and true values for motion in the LR, AP and SI directions are all within 0.6 mm and results for both patients are accurate within 1 mm in 3D Euclidean space. Based on these results and validation studies in the literature with other treatment CT imaging modalities, our implementation of the automatic prostate localization algorithm can accurately be applied to MVCT images.

**Table 1:** Comparison of actual prostate motion as determined from the positions of implanted localization seeds and measured prostate motion established with the automatic prostate localization algorithm.

	*Actual Prostate Motion*	*Measured Prostate Motion*	*Difference*
Patient 1			
LR	0.2 mm	0.3 mm	0.1 mm
AP	−2.6mm	−2.0mm	0.6 mm
SI	2.5 mm	2.0 mm	−0.5mm
Patient 2			
LR	−0.5mm	−0.7mm	−0.2mm
AP	−1.9mm	−1.9mm	0.0 mm
SI	0.7 mm	0.9 mm	0.2 mm

### B. dependence on planning CT contour delineation

**Figure 2:**
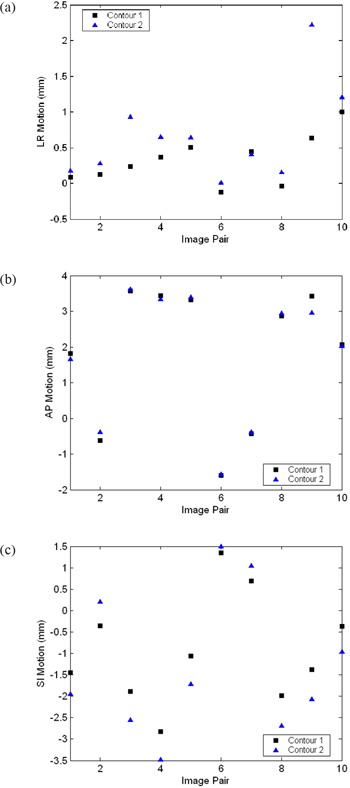
Prostate motion as measured by the automatic prostate localization software using two different input planning CT contours. Analysis was performed for ten randomly selected fractions from a single patient: (a) left‐right (LR) motion, (b) anterior‐posterior (AP) motion, (c) superior‐inferior (SI) motion.

After redrawing of the prostate contour on a single patient planning CT, the second radiation oncologist‐delineated contour was input into the automatic prostate localization software and the multi‐start procedure was repeated for ten randomly selected MVCT images. Registration results from each planning CT contour were analyzed by calculating the absolute differences in prostate motion values produced by the two prostate localization procedures. The prostate motion values in the LR, AP, and SI directions for each input contour for all ten fractions are illustrated in Fig. 2. Average (±standard deviation) differences were 0.4±0.5mm,0.1±0.1mm, and 0.6±0.2mm in the LR, AP, and SI directions, respectively, demonstrating that the automatic prostate localization software has little dependence on intraobserver contouring discrepancies. For reference, the location of the centroid position in each contour differed by 0.8 mm in the LR direction, 0.5 mm in the AP direction, and 1.9 mm in the SI direction. Volumes were 36.7 cc and 37.3 cc, respectively.

### C. Prostate positioning errors

**Figure 3:**
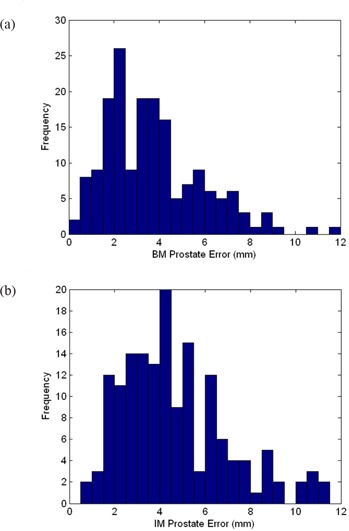
Frequency histograms for 3D prostate positioning errors when daily patient repositioning is based on (a) bone matching (BM) and (b) image matching (IM).

Upon completion of the bone matching, image matching, and prostate localization multistart procedures for each image pair, the prostate positioning errors that would have occurred if daily patient repositioning was based on BM or IM were evaluated. Registrations that failed the multistart procedure twice were excluded from analysis. For example, if image matching for a particular MVCT image failed, the IM prostate positioning error could not be calculated. Histograms for the BM and IM prostate positioning errors for all analyzed patient fractions are displayed in Fig. 3. In addition, mean and maximum 3D positioning errors for each individual patient are listed in Table 2. Also included are standard deviation values, which represent the range in positioning errors from fraction to fraction associated with each matching method. Results across the board demonstrate that daily prostate positioning using bone matching is superior to using image matching. For 157 treatment fractions, the mean prostate positioning error from image matching was 4.6 mm, with a standard deviation of 2.3 mm. The maximum value was 11.5 mm. In the 175 fractions that the prostate positioning error from bone matching was calculated, the mean error was 3.7 mm, with a standard deviation of 2.1 mm. The maximum value was found to be 11.8 mm. Results were also analyzed in terms of each of the translational directions by calculating the percentage of fractions in which BM and IM would result in a prostate positioning error greater than 3 mm and greater than 5 mm in each direction. The 3 mm and 5 mm results are given in Tables 3 and 4, respectively.

**Table 2:** Statistics for prostate positioning errors based on bone matching (BM) and image matching (IM).

		*Bone Matching*				*Image Matching*		
*P*	*n*	*Mean (mm)*	*σ (mm)*	*Max (mm)*	*n*	*Mean (mm)*	*σ (mm)*	*Max (mm)*
1	19	3.4	1.8	7.1	18	4.1	1.6	7.3
2	23	3.9	1.8	8.6	22	5.1	2.0	10.0
3	24	5.4	2.6	11.8	20	6.9	2.8	11.5
4	24	3.1	1.8	7.7	23	3.5	2.1	10.8
5	18	3.4	2.2	8.6	16	4.1	2.1	8.6
6	22	2.4	1.6	7.0	19	3.2	1.4	6.1
7	21	4.9	1.9	9.1	18	6.0	1.5	9.0
8	24	3.2	1.8	8.3	21	4.1	2.4	10.8

P=patient number;n=number of fractions analyzed

**Table 3:** Percentage of registrations that resulted in a prostate positioning error ≥3mm in each of the translational directions.

	*LR*	*AP*	*SI*
Bone Matching (BM)	4%	37%	25%
Image Matching (IM)	7%	39%	34%

**Table 4:** Percentage of registrations that resulted in a prostate positioning error ≥5mm in each of the translational directions.

	*LR*	*AP*	*SI*
Bone Matching (BM)	0%	11%	7%
Image Matching (IM)	0%	17%	14%

### D. Interfraction prostate motion

**Figure 4:**
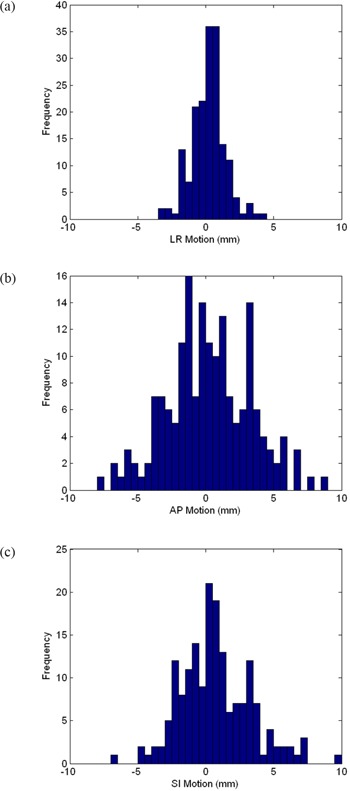
Interfraction prostate motion histograms for 175 treatment fractions: (a) left‐right (LR) motion, (b) anterior‐posterior (AP) motion, (c) superior‐inferior (SI) motion.

Once the multistart procedures have been completed, evaluation of the offsets between prostate localization and bone matching in the LR, AP and SI directions provides a measure of the daily interfraction prostate motion. The distribution of values is shown in Fig. 4 and the interfraction prostate motion statistics are given in Table 5. The observed standard deviations were 1.2 mm in the LR direction, 3.1 mm in the AP direction, and 2.6 mm in the superior‐inferior direction. Mean values were sub‐mm in each of the three directions, suggesting there are no systematic effects present.

**Table 5:** Interfraction prostate motion statistics for entire cohort. Negative values correspond to motion superiorly, anteriorly, and to the left.

*Direction*	*Mean (mm)*	*σ (mm)*	*Range (mm)*
Left/right (LR)	0.2	1.2	−3.3 to 4.2
Anterior/posterior (AP)	0.2	3.1	−7.6 to 8.7
Superior/inferior (SI)	0.7	2.6	−6.9 to 10.7

## IV. DISCUSSION

Of the 204 multistart procedures performed for BM, IM, and prostate localization, the acceptance rates were 97%, 90%, and 89%, respectively. The acceptance rate for prostate localization requires further discussion. For temporal reasons and to reduce gonadal dose, care was taken to minimize the extent of daily MVCT image acquisition inferior to the prostate gland. As a result, numerous MVCT images used in this study did not contain the entire prostate. If only the multistart prostate procedures in which the entire prostate is contained in the MVCT image are considered, the acceptance rate increases to 95%.

We have demonstrated that significant prostate positioning errors will occur when daily patient repositioning in the Hi·Art II system is performed based on bone matching and image matching. To put the results into perspective, consider that if during daily treatment, patients were repositioned based on the registration of MVCT and planning CT images using the standard automatic image matching algorithm used in this study, on average the prostate would be 4.6 mm away from where it should be during each treatment fraction. On the other hand, if repositioning the patient based on bone matching, the average daily prostate misplacement would be reduced to 3.7 mm. In terms of the translational directions, IM would lead to prostate positioning error ≥3mm in any of the LR, AP or SI directions in 62% of treatment fractions. The corresponding value for BM is 51%. Analogous values for positioning errors ≥5mm are 29% and 17% for IM and BM, respectively. These results suggest that location of the prostate is better correlated with pelvic bony anatomy as opposed to the pelvic anatomy as a whole. One possible explanation is that prostate positioning errors associated with bone matching are solely dependent on the motion of the prostate with respect to rigid anatomy, whereas in image matching, the overlap of amorphous structures such as the outer patient contour may also contribute to positioning errors.^(14)^ As such, given the choice of performing daily patient repositioning based on automatic image matching or automatic bone matching, the latter should be chosen.

Results for all eight patients are consistent with the cohort data in that errors are greater for image matching than bone matching. In particular, there were three patients in which the mean value for image matching error was greater than 5 mm. The source of the large value is different for each patient; however, they can all be attributed to the fact that the prostate gland moves in response to rectal and bladder filling.^(^ ^20^ ^,^ ^21^ ^)^ The standard treatment protocol for prostate patients at our clinic is for a full bladder and empty rectum. Any deviation from this standard during planning CT image acquisition or during treatment can lead to the prostate being in a different location in MVCT images than in planning CT images. Patient 2, who had a mean image matching error of 5.1 mm had a significant amount of intestinal gas present during the planning CT that was not present in the majority of daily MVCT images. The treatment protocol was not fully satisfied for Patient 7, either, as the patient's bladder was not entirely full during planning CT acquisition and as a result, the bladder was significantly larger in the majority of MVCT images. The largest mean deviation occurred for Patient 3 and the explanation is slightly different. The patient had a full bladder and empty rectum during planning CT acquisition; however, the patient had large quantities of bowel gas during the majority of pretreatment MVCT acquisitions, and presumably during treatment as well.

In the treatment of the patients investigated in this study, the prostate and seminal vesicles were taken to be the clinical target volume (CTV) and a margin of 7 mm posterior and 10 mm around the remainder of the CTV defined the planning target volume (PTV). Although observed offset values tend to be well within the extent of these margins, these offsets are clinically significant for a couple of reasons. Firstly, the entire PTV is planned to receive the prescription dose. If after patient repositioning based on IM the prostate location is off by the mean value of 4.6 mm (although the entire CTV will still receive the prescription dose), there is a high probability that part of a surrounding critical structure will also receive that prescription dose. In addition, studies have shown that increasing the prescription dose in prostate radiotherapy leads to improved cure rates^(22)^ and as doses continue to escalate, margins will need to be reduced accordingly in order to minimize dose to the surrounding critical structures. With reduced margins, prostate localization errors must be considered when performing automatic registration‐based daily positioning in order to prevent the CTV from being underdosed.

As mentioned previously, Langen et al.^(8)^ investigated manual registration techniques for daily prostate positioning on the Hi·Art II system and found that without the use of implanted fiducials, prostate errors greater than 3 mm in any one direction were common. We have shown that automatic registration methods based on bone matching and image matching also lead to daily prostate positioning errors. Although errors have been quantified for both manual and automatic methods, a statement advocating one technique over the other requires evaluation using the same cohort of image data. Regardless of the positioning method, prostate positioning errors are not dosimetrically acceptable in standard clinical practice. For example, Wong et al.^(6)^ investigated IMRT patients treated on a Siemens Primus linear accelerator and found that for a typical prostate case, failure to correct for a 10 mm posterior prostate shift results in a drop in CTV dose coverage from 95%–107% to 71%–100%. Further research is required to determine the extent of the dosimetric implications of prostate positioning errors for Hi·Art II patients. The only way to truly eliminate prostate positioning errors when performing daily patient repositioning‐based image registration is to ensure that the daily registration results in accurate prostate overlap. One solution would be to have accurate automatic prostate localization tools incorporated into the Hi·Art II system. Further research is required before the algorithm used in this work can be implemented clinically on a daily basis. The multistart procedure and the highly stringent convergence criterion used in our implementation would not permit for registration to be performed in clinically allowable times during treatments.

In 2001, a review of all the interfraction prostate motion studies in the literature was compiled by Langen and Jones.^(20)^ Standard deviations ranged from 0.7 mm to 1.9 mm for LR motion, 1.5 mm to 4.1 mm for AP motion, and 1.7 mm to 4.5 mm for SI motion. Not only does our data fit in the middle of the range of values in the reported literature, our results also follow the common trend that interfraction prostate motion is greater in the AP and SI directions than in the LR direction. More importantly, we have shown that interfraction prostate motion can be measured in Hi·Art II prostate patients using automated registration techniques. This allows for a number of potential research opportunities.

## V. CONCLUSIONS

We have quantified the prostate positioning errors that would occur if daily patient positioning of eight research patients treated on the Hi·Art II system was based on automatic image matching and automatic bone matching. Clinically significant errors ranging up to 11.8 mm were observed. If the assumption is made that the prostate gland should be in the same location during each treatment fraction, automatic IM and automatic BM would have led to average prostate positioning errors of 4.6 mm and 3.7 mm, respectively. Further work is required to determine the dosimetric and biological consequences of these positioning errors, not only for the target volume, but also the surrounding dose sensitive structures. Ideally, an accurate automatic prostate localization algorithm should be implemented during daily treatment on the Hi·Art II system. The algorithm used in this work is a candidate; however, the code must be modified in order for it to operate quickly enough to be practical for clinical use. Whether or not such timesaving modifications will compromise the algorithm's accuracy remains to be seen. As such, further research is required before the algorithm used in this work can be implemented clinically.
